# The Book World of Medicine and Science

**Published:** 1901-03-02

**Authors:** 


### The Book World of Medicine and Science.

The Metropolitan Hospitals and Vivisection. A Guide for the Charitable in the Disposition of their Gifts and Bequests. (London: The National Anti-Vivisection Society. 1901.)

The latest performance of the Anti-Vivisection Society is the issue of this so-called guide for the charitable, which is in fact nothing more or less than a malicious attack upon the great hospitals of London, especially those which have attached to them Medical Schools in which pathological and physiological laboratories exist. In the list of hospitals here given Mr. Coleridge prints in red ink those which "have vivisectors on their staffs, and have attached to them Medical Schools licensed for vivisection;" in italics those that "have licensed vivisectors on their staffs, but have no Medical Schools licensed for vivisection attached to them;" and in ordinary type those that "are now entirely free from any connexion with vivisection." It is to the latter, of course, that the charitable and humane people are asked to give, but it being a somewhat sorry list of small and unimportant charities, the author of this precious document has had to eke it out by the insertion of the rate-supported fever hospitals of the Metropolitan Asylums Board! The absurdity of including in a list for the guidance of the charitable a crowd of institutions which are supported by poor-law funds is sufficiently apparent. But does not Mr. Coleridge know that these very institutions, which he selects as "entirely free from all connexion with vivisection," are themselves the great supporters of vivisection? According to the last report of the Metropolitan Asylums Board, no less than 7,038 cases of diphtheria were treated in their hospitals with antitoxin during the year 1899—antitoxin, all of which was produced by infecting horses with diphtheria poison, and then bleeding them again and again to obtain the serum of their blood. This is vivisection if anything is, and it is done daily to provide material for the use of those very hospitals which Mr. Coleridge places on his list as "free from all connexion with vivisection." The fact is that the publication of this list is merely an attack on certain selected hospitals which honestly and openly do the best they can for their patients, while other institutions, which make use of the benefits derived from vivisection without actually being attached to Medical Schools where the work is done, are included in the list of the blessed.

Memoir of James Macartney, M.D., F.R.S. By Prof. Alexander Macalister, M.D., F.R.S. (Hodder and Stoughton.)

Professor Macalister has compiled an interesting memoir of Dr. James Macartney, to whose ability as a pioneer in anatomical teaching the Dublin School of Medicine owed the prestige it enjoyed during the earlier half of last century. James Macartney was born at Armagh in 1770, his forbears having migrated from Scotland. His school career was short and interrupted, and at the age of sixteen he was placed in the office of a linen merchant at Newry, where he remained for a year. On the death of his father he returned home to help his three brothers in farming the family land. It is curious to notice that his thoughts first turned to surgery as a profession after meeting with a disappointment in love in 1794. He says in his diary, "I thought surgery would harden my heart, as it has others: in this I have been disappointed." Later on, however, he renewed his addresses to this lady, a Miss Ekenhead, and this time they were accepted, and she became his wife in 1790. After his marriage he went to England to pursue his medical studies, and in course of time became a dresser at Guy's Hospital, and was afterwards appointed Lecturer on Comparative Anatomy at St. Bartholomew's Hospital, where he inaugurated the first systematic course of biological lectures ever given in the United Kingdom. Soon afterwards he was appointed surgeon to the Royal Radnor Militia, but, in 1812, the disbandment of that force left him free to apply for the then vacant professorship of anatomy at Dublin. On obtaining the appointment Dr. Macartney immediately set about the series of reforms which were eventually to place the anatomical teaching at Dublin far above that in any other contemporary school in Great Britain. He suffered the common lot of pioneers in meeting with discouragement and opposition from the hands of his colleagues. Eventually, however, he obtained a grant of money for the erection of much needed new class-rooms and laboratories. The architect chosen by the Board seems to have been grossly incompetent, and it is amusing to read that Macartney's indignation so got the better of him on one occasion during the construction of the buildings, that "the architect struck Macartney with his cane, whereupon Macartney retaliated by breaking his umbrella over the architect's head." One of the many difficulties which confronted Macartney in his work was the obtaining of bodies for his research work and demonstration lectures on surgery. It was the time of the Burke and Hare atrocities, and, like all dissecting-rooms of that day, those of Macartney were entirely dependent for a supply of "subjects" on the craft of "resurrectioners." At one time popular fury in Dublin gravely threatened the safety of the Professor and his laboratories, but in 1834 the Act was passed which legalised the dissection of the bodies of unclaimed paupers, and a serious obstruction and annoyance was thus removed from Macartney's path. From that time onward, however, he was constantly subjected to petty and malicious interference on the part of his professional brethren, and in 1837 he decided to resign his professorship. He died in 1843. The memoir is interestingly written, and should appeal to a wide area of readers in the Medical world.

### NOTES ON BOOKS.

The Journal of the Sanitary Institute which has just been issued contains a long article by Professor A. Celli, of Rome, on "Researches on the Propagation of Malaria and the Precautions to be adopted in Unhealthy Districts." He describes the life history of the Anopheles, and sets out a series of corollaries for the hydraulic engineer, for the sanitary engineer, and for the agriculturist, in which are detailed the steps that can be taken to prevent the multiplication of the Anopheles. The concluding portion of the paper gives instructions for the self-protection of labourers in malarious places. The paper is illustrated from some of Professor Celli's photographs showing methods of protecting houses and shelters.

